# Assessing the consequences of quarantines during a pandemic

**DOI:** 10.1007/s10198-021-01310-3

**Published:** 2021-05-06

**Authors:** Rikard Forslid, Mathias Herzing

**Affiliations:** 1 Stockholm University, Stockholm, Sweden; 2 CEPR, London, UK

**Keywords:** Pandemics, Quarantine, SEIR-model, COVID-19, Economic consequences, D42, D62, H10, I18, L10

## Abstract

This paper analyzes the epidemiological and economic effects of quarantines. We use a basic epidemiological model, a SEIR-model, that is calibrated to roughly resemble the COVID-19 pandemic, and we assume that individuals that become infected or are isolated on average lose a share of their productivity. An early quarantine postpones but does not alter the course of the pandemic at a cost that increases in the duration and the extent of the quarantine. For quarantines at later stages of the pandemic there is a trade-off between lowering the peak level of infectious people on the one hand and minimizing fatalities and economic losses on the other hand. A longer quarantine dampens the peak level of infectious people and also reduces the total number of infected persons but increases economic losses. Both the peak level of infectious individuals and the total share of the population that will have been infected are U-shaped in relation to the share of the population in quarantine, while economic costs increase in this share. In particular, a quarantine covering a moderate share of the population leads to a lower peak, fewer deaths and lower economic costs, but it implies that the peak of the pandemic occurs earlier.

## Introduction

This paper analyzes the epidemiological and economic effects of quarantines. More specifically, our focus is on how the timing, duration and extent of a quarantine impact on the dynamics of a pandemic as well as on economic losses.

In the absence of a vaccine or efficient drugs, countries have had to adopt old-fashioned practices to combat the COVID-19 pandemic. One such policy is the use of quarantines. A complete quarantine will eventually bring down the number of newly infected persons to zero (as has been achieved at the regional level in e.g. China). However, as a large part of the population remains susceptible, complete lockdowns might have to be followed up with further restrictions and extensive tracing.

Here, the focus is on quarantines that are less than complete, such as those implemented by many European countries during the last year. These quarantines imply that fewer individuals will be infected at the peak of the infection and that the peak will occur later in time. Both these effects are important in order to prevent the health care system from being completely overwhelmed. However, quarantines have substantial economic costs, as production closes down when workers are confined to stay at home.

When the COVID-19 pandemic struck at the beginning of 2020, countries adopted very different strategies regarding the use of quarantines. China implemented an almost complete lockdown in Wuhan and in some other cities in the Hubei province on 23 January; the lockdown officially ended on 8 April after the number of daily new cases reached zero. European countries also introduced quarantines of various degrees of restrictiveness. Italy, which was hit very hard by the COVID-19 infection early on, implemented a very restrictive quarantine. For instance, in Codogno (pop. 16,000), one of the most affected towns, police cars blocked roads into and out of the quarantined area and erected barriers. In many countries schools and most shops were closed nationwide, and gatherings of only limited numbers of people were allowed in public spaces. Denmark was among the first European countries to introduce lockdown measures, starting on 13 March 2020; these were eventually lifted gradually before being re-implemented during the second wave of the Corona pandemic in the fall of 2020. In contrast, Sweden did not impose any quarantine, kept primary schools and pre-schools open and allowed public gatherings of up to 50 people during the entire first wave of the COVID-19 pandemic; however, the Swedish authorities instructed people to work from home if possible and not to travel unnecessarily.

In this paper we analyze the effects of quarantines of different extents and durations that are imposed at different points during a pandemic. We use a basic epidemiologic model, a SEIR-model, that is parameterized to roughly resemble the COVID-19 pandemic.^1^ As in Atkeson [3] we assume that individuals that become infected on average lose a share of their productivity, and we also assume that quarantined individuals on average incur productivity losses. However, our qualitative results do not depend on the assumed values of productivity losses. Our main findings can be summarized as follows.

1. The implementation of an early quarantine will essentially postpone but not alter the course of the pandemic at a cost that increases in the duration and the extent of the quarantine.
2. For quarantines starting at later stages of the pandemic there is a trade-off between lowering the peak level of infectious people on the one hand and minimizing fatalities and economic losses on the other hand. A quarantine implemented when the number of infectious persons starts increasing rapidly is optimal if the main goal is to reduce the peak level of infectious people. A starting day just before the peak of infectious people is reached is optimal if the aim is to minimize fatalities and economic losses.
3. There is a trade-off between economic costs and health outcomes in terms of the duration of a quarantine. A longer quarantine either postpones the peak (if it is implemented relatively early) or dampens the peak and reduces the total number of infected people and deaths (if it starts at a later stage of the pandemic), but implies higher economic losses.
4. The peak level of infectious people as well as the total share of the population that will have been infected are U-shaped in relation to the extent of a quarantine (the share of the population in quarantine). A quarantine of moderate extent, covering around one third of the population, leads to a lower peak, fewer deaths and lower economic costs than a more complete lockdown. However, it implies that the peak of infectious people occurs earlier.

Several recent papers analyze the implications of the policy response in relation to the COVID-19 pandemic. Dewatripont et al. [9] discuss how to best use testing. Hall et al. [11] analyze the optimal trade-off between consumption losses and pandemic deaths. Jones et al. [13] studies the interaction of private and public mitigation efforts. Other policy options are discussed in Baldwin and Weder di Mauro [4] and Baldwin and Weder di Mauro [5]. More closely related to us, a number of recent papers specifically analyze the consequences of isolation enforcement. Anderson et al. [2] discuss how mitigation policies will affect the COVID-19 pandemic. Casares et al. [8] calibrates a dynamic model for the Spanish economy. The study shows how isolation or quarantines slow down the speed of the contagion and reduces the numbers of infected and dead. However, they do not consider the economic effects of quarantines. Piguillem et al. [15] calibrate a SEIR- model to Italian data, and calculate the optimal path of a quarantine for different functional forms of the planner’s utility function. Similarly Alvarez et al. [1] and Gonzalez-Eiras and Niepelt [10] employ optimal control theory to determine the optimal path of a quarantine that can be continuously varied. Zou et al. [16] calibrate a phenomenological logistic model to Chinese data on COVID-19. They find that stringent measures (quarantines) can suppress the infection within 60 days.

We do not calibrate our model to any particular country and do not use control theory to pin down an optimal path of isolation. Our purpose is instead to try to shed light on some of the underlying trade-offs between economic and health outcomes when a quarantine is implemented.

## The model

We employ a SEIR-model similar to Atkeson [3]. There are five categories of individuals: susceptible persons (*S*) who have never been exposed to the virus; exposed persons (*E*) who carry the virus, but are not yet infectious; infectious persons (*I*); recovered persons (*R*) who are no longer infectious and are assumed to have become immune to the virus; and deceased persons (*D*). A susceptible individual becomes infected by infectious individuals at the rate $\beta I$. Exposed persons become infectious at rate $\varepsilon$. Infectious persons recover at rate $\gamma$ and die at rate $\delta$. The dynamics of the SEIR-model can be summarized as follows:

$$\begin{aligned} \overset{.}{S}&=-\beta SI, \\ \overset{.}{E}&=\beta SI-\varepsilon E, \\ \overset{.}{I}&=\varepsilon E-\gamma I-\delta I, \\ \overset{.}{R}&=\gamma I, \\ \overset{.}{D}&=\delta I. \end{aligned}$$

For simplicity it will be assumed that *S*, *E*, *I*, *R* and *D* represent shares of the population, i.e. $S(t)+E(t)+I(t)+R(t)+D(t)=1$ at any point in time *t*.

Most countries have responded to the present Corona pandemic by imposing different types of quarantines, covering large parts of the population. In the context of the present model a quarantine would cover a constant share *q* of susceptible, exposed, infectious and recovered individuals over a certain period. The quarantined population would thus consist of the shares $S_{Q}$, $E_{Q}$, $I_{Q}$ and $R_{Q}$. For simplicity we assume that there is no transmission of the virus among the quarantined population, i.e. $S_{Q}(t)$ remains constant during the quarantine. In reality, the virus could be transmitted within quarantined families; allowing for a small rate of transmission among the quarantined population would not alter our analysis qualitatively. Quarantined exposed individuals become infectious at rate $\varepsilon$, and quarantined infectious individuals recover at rate $\gamma$ and die at rate $\delta$. The dynamics during the quarantine can thus be summarized as follows:

$$\begin{aligned} \overset{.}{S}&=-\beta SI, \\ \overset{.}{E}&=\beta SI-\varepsilon E, \\ \overset{.}{I}&=\varepsilon E-\gamma I-\delta I, \\ \overset{.}{R}&=\gamma I, \\ \overset{.}{D}&=\delta I+\delta I_{Q} \\ \overset{.}{S_{Q}}&=0, \\ \overset{.}{E_{Q}}&=-\varepsilon E_{Q}, \\ \overset{.}{I_{Q}}&=\varepsilon E_{Q}-\gamma I_{Q}-\delta I_{Q}, \\ \overset{.}{R_{Q}}&=\gamma I_{Q}. \end{aligned}$$

After the quarantine has been terminated, the quarantined individuals join their corresponding groups, e.g. $E_{Q}$ is added to *E*. Here, we do not account for quarantines that are introduced and lifted in steps. In reality, a government can vary the extent of a quarantine and let smaller groups of people return to normal life. However, there are infinitely many possibilities for implementing a quarantine. To keep our analysis transparent we only consider quarantines that take place once for a certain duration and covering a constant share of the population.

To assess the implications of a quarantine we will focus on the following measures:

(i) The peak of the share of infectious individuals $I_{Peak}$. From a public health perspective it is desirable to dampen the maximum number of infected persons.
(ii) The day $t(I_{Peak})$ when the peak of the share of infectious individuals occurs. For the public health authorities a later day is preferable, because it allows hospitals to be better prepared.
(iii) The share of the population that will have been infected and survived 1 year after the start of the pandemic, which is measured by the share of recovered individuals on day 365 of the pandemic *R*(365); the share of deceased persons is obviously proportional to that number. To keep the number of infected and hence, deceased individuals low is one important objective.
(iv) The economic output during 1 year *Y*, from day 0 to day 365. In the absence of the pandemic it is assumed that productivity is 1 per individual and day, i.e. normalized total output would be 366 for the entire population. It is assumed that the productivity of susceptible, exposed and recovered individuals is 1 if there is no quarantine, whereas those in quarantine will have an average productivity $b=0.5$, reflecting the fact that some individuals, e.g. individuals employed as manual workers, may have close to zero productivity, whereas other professions or tasks are easier to perform from home. Likewise infectious persons either have no or only mild symptoms or are sick at home or need costly treatment in a hospital. It is assumed that their average productivity is reduced to a fraction $a=0.5$. The productivity parameters determine the economic impact of the quarantine, but they do not affect the dynamic properties of the model.

Normalized total output at any day *t* is given by

$$\begin{aligned} Y(t)& = {} S(t)+E(t)+R(t)+aI(t)\\&+b\left[ S^{Q}(t)+E^{Q}(t)+R^{Q}(t)+aI^{Q}(t)\right] . \end{aligned}$$

To assess the economic consequence of the pandemic, $Y=\sum \nolimits _{t=0}^{365}Y(t)$ will be measured. It is thus implicitly assumed that the pandemic only has short-term consequences in the sense that it only leads to lost output due to illness and, possibly, a quarantine. Long-term structural effects are, therefore, not accounted for. Once the pandemic is over, the economy reverts to the status quo ante.

## Simulations

We do not intend to calibrate the infection dynamics to any particular country or case, but we do have the COVID-19 pandemic in mind, and we have, therefore, chosen parameter values that have been suggested for this infection. The average incubation period is five days, but it seems that you can spread the infection two days before that.^2^ We, therefore, set $\varepsilon =\frac{1}{3}.$ We also assume that it takes on average two weeks to recover, implying that $\gamma =1/14$, and that $0.1\%$ of infectious persons die, i.e. $\delta =0.001/14$.^3^ Finally, we have $\beta =0.2$ in the base case, which reflects the speed of the spread of the pandemic without a quarantine.^4^

### Base case: no quarantine

**Fig. 1:**
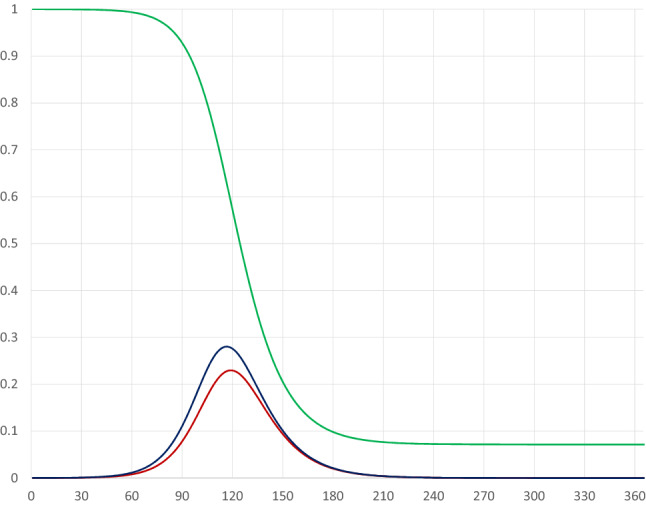
Pandemic dynamics in the absence of a quarantine where the red, blue and green curves represent *I*(*t*), $I(t)+E(t)$ and $I(t)+E(t)+S(t)$

The base case scenario has no quarantine. Figure 1 illustrates the pandemic dynamics during the course of 1 year given our parameter values.

The horizontal axis measures days since the start of the pandemic, while the vertical axis measures shares of the population. The red curve represents the share of infectious individuals, the blue curve represents the share of infectious plus exposed individuals, and the green curve represents the share of infectious, exposed and susceptible individuals. That is, recovered and deceased individuals are represented by the area above the green curve; deceased people represent only a tiny fraction ($0.1\%$) of these.

Assuming that at the start of the pandemic $0.01\%$ had been exposed to the virus (i.e. $S(0)=0.9999$), the peak of infectious individuals would occur on day 118 and represent 23 percent of the population.^5^ Moreover, a year after the pandemic started almost $93\%$ would belong to the category of recovered (and possibly resistant) individuals, implying a share of $7\%$ still being susceptible. Furthermore, output would be reduced from 366 to 359.28, representing a fall of $1.84\%$, due to the pandemic.^6^

Buss et al. [7] report the consequences for the Brazilian city Manaus of having let the pandemic run its course without any effective mitigation. In May 2020 the COVID-19 incidence peaked in Manaus with health services shattered and very high fatality numbers (4.5-fold excess mortality). Despite the total share of infected people rising to 76 percent in October, herd immunity may still not have been achieved there.

### Introducing a quarantine

When assessing the effects of a quarantine several factors are of interest:

(i) timing, i.e. the start of the quarantine;
(ii) the duration of the quarantine;
(iii) the extent of the quarantine, i.e. how large a share of the population is covered.

Below we present results from simulations to illustrate the importance of these factors.

#### Timing of the quarantine

**Fig. 2:**
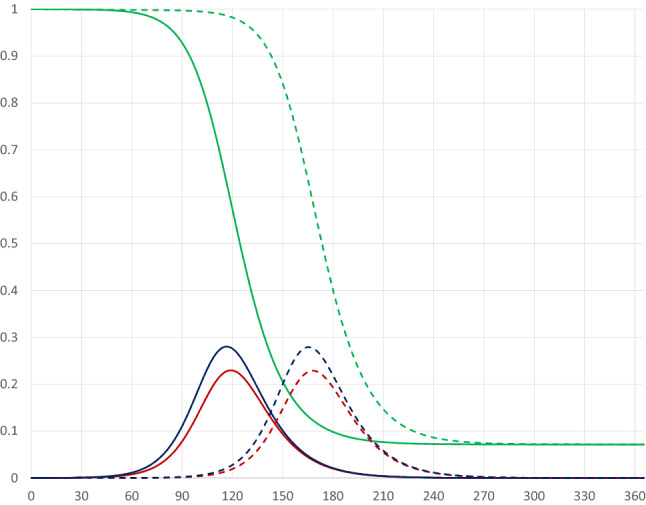
Pandemic dynamics with no quarantine (solid curves) and with a 30 day quarantine covering 80 percent of the population starting on day 30 (dashed curves)

Figure 2 illustrates the pandemic dynamics in the absence of a quarantine (solid curves, the same as in Fig. 1) and for a thirty-day quarantine covering 80 percent of the population starting on day 30 of the pandemic (dashed curves). At early stages of the pandemic the starting date of the quarantine has almost no effect on the dynamics; a later starting date in the early phase of the pandemic (i.e. before the number of infectious persons has started to increase rapidly) will simply postpone it.

**Fig. 3:**
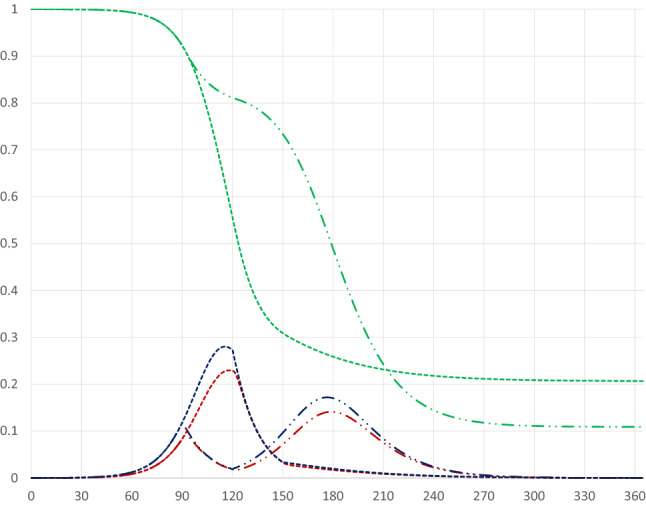
Pandemic dynamics for 30 day quarantines covering 80 percent of the population starting on days 90 (dashed-dotted curves) and 120 (dotted curves)

If the quarantine starts at a stage when the share of infectious individuals is increasing rapidly, the pandemic dynamics are affected differently, as illustrated by a quarantine starting on day 90 in Fig. 3 (dashed-dotted curves). In this case there will be a double-peak in the share of infectious individuals, as its rise is stopped, but it starts increasing again after the quarantine has been terminated.^7^,^8^ If the quarantine starts later, just before or after the peak of the share of infectious individuals has been reached, there will be a faster drop from the peak, as illustrated by the pandemic dynamics for a quarantine starting on day 120 (dotted curves).^9^ Compared to the base case quarantines starting at later stages of the pandemic lead to fewer infected persons and, therefore, to fewer deaths. The quarantine starting on day 120 leads to fewer being infected than the quarantine starting on day 90, but at the cost of a higher peak level of infectious individuals.

**Fig. 4:**
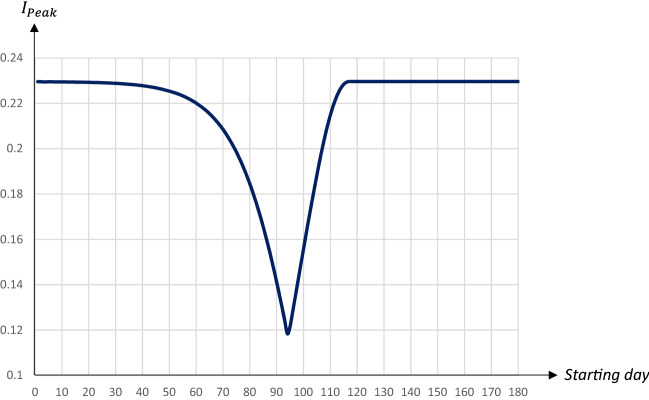
The peak level of infectious people in relation to the starting date of a 30 days quarantine covering 80 percent of the population

**Fig. 5:**
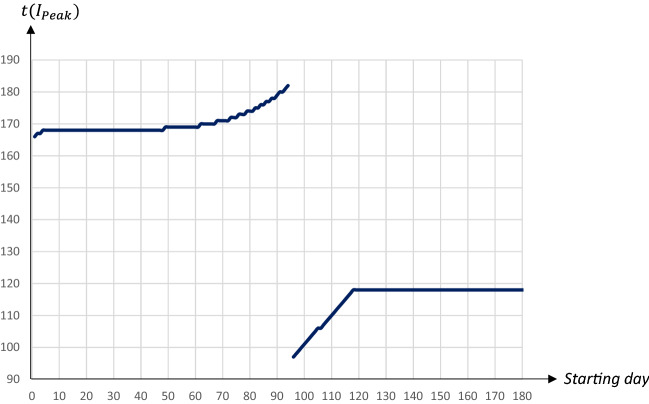
The day of the peak level of infectious people in relation to the starting date of a 30 days quarantine covering 80 percent of the population

Figures 4 and 5 illustrate how the peak level of infectious individuals and the day when this peak level is reached are affected by the timing of a 30-days quarantine covering 80 percent of the population; the horizontal axis measures the day of the pandemic when the quarantine starts.

The perhaps most striking result is that there is a U-shaped relationship between the starting day of the quarantine and the maximum share of infectious individuals. An early quarantine primarily postpones the infection (see Fig. 2); once lifted the infection runs its course, and since there are still many susceptibles in the population the peak will be high. A late quarantine, just before or after the peak of the infection has passed, has no effect on the level of he peak (see Fig. 3). The peak of the infection is, therefore, mostly reduced by a quarantine starting when the number of infectious individuals increases most rapidly. This results in a double peak in the share of infectious persons, leading to a drop in the peak day as the first peak becomes larger than the second peak.

In the example above, a quarantine starting on day 94 seems optimal in terms of reducing the peak level of infectious individuals; it decreases to less than 12 percent from almost 23 percent in the absence of a quarantine. This is a remarkably stable result; although peak levels obviously depend on the duration and the extent of a quarantine, those starting around this date generally yield the lowest peak levels.^10^

**Fig. 6:**
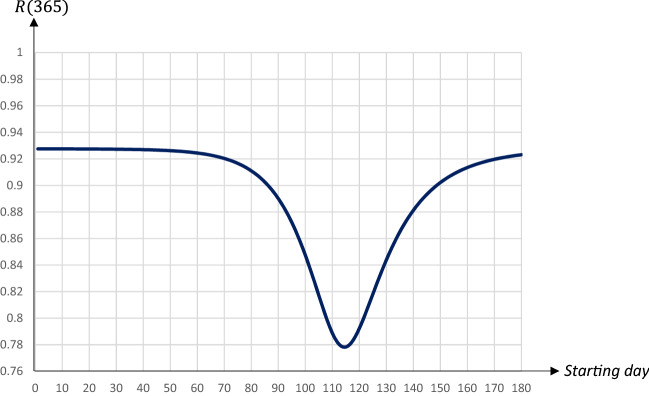
The share of population that has recovered one year after the start of the pandemic in relation to the starting date of a 30 days quarantine covering 80 percent of the population

**Fig. 7:**
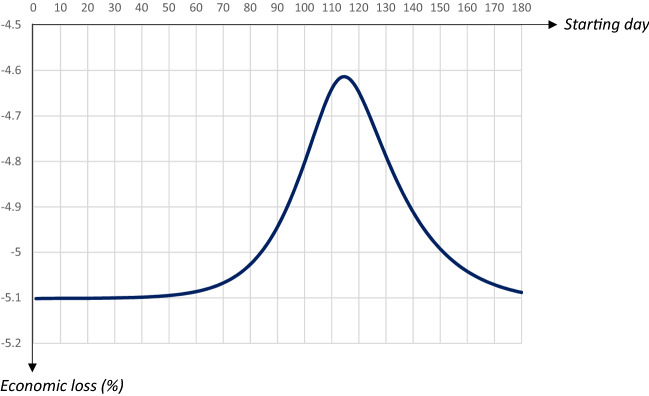
Economic losses in relation to the starting date of a 30 days quarantine covering 80 percent of the population

Figures 6 and 7 show the share of population that has recovered after the pandemic as well as the economic losses with respect to the starting date.

There is a U-shaped relationship between the total number of individuals that have been infected and recovered (and hence, also the total number of deceased individuals) and the starting day of the quarantine. The lowest level is reached for a quarantine starting on day 114. In this case the share of the population that will have been infected and survived will be less than 78 percent, as compared to almost 93 percent in the absence of a quarantine. It is worth noting that herd immunity would be achieved for any 30 days quarantine covering 80 percent of the population, regardless of the starting day.^11^

The relationship between the economic loss and the starting date is also U-shaped, with economic losses minimized for a quarantine starting on day 115. Interestingly there seems to be no trade-off between economic losses and averting fatalities. The total number of deaths and the economic losses are both minimized when the quarantine is implemented around days 114–115. Thus, to keep fatalities as well as economic losses low it seems optimal to postpone a quarantine to just before the share of infectious individuals reaches its peak. Also this result is stable; obviously levels depend on the duration and extent of a quarantine, but the general pattern is similar.^12^ The downside, however, is that this policy does little to reduce the peak, which would make it hard to implement due to the limited capacities in the health care system.

To summarize, there is a trade-off between lowering the peak level of infectious people on the one hand, and reducing fatalities as well as economic losses on the other hand. If the main goal is to lower the $I_{Peak}$-level an earlier quarantine starting day within this time frame is preferable, while a later starting day would be optimal if the main goal is to reduce fatalities and/or economic losses. An implication of this is that a high capacity for intensive care treatment in the health care system implies that the government can chose a strategy that leads to both fewer deaths and lower economic losses.

#### Duration of the quarantine

We now turn to the effect of the duration of a quarantine. We simulate quarantines that cover 80 percent of the population. As demonstrated in the previous section, the timing of a quarantine impacts crucially on the pandemic dynamics. To analyze the effects of a quarantine’s duration we, therefore, distinguish between those implemented early and those started later, when the share of infectious individuals starts taking off.

**Fig. 8:**
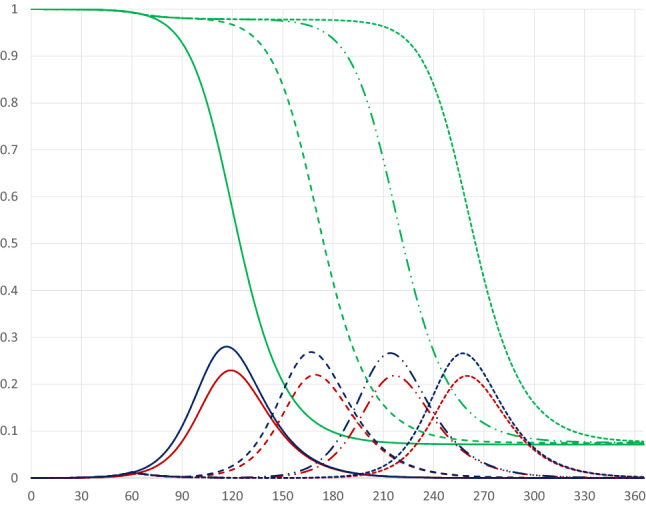
Pandemic dynamics for quarantines covering 80 percent of the population starting on day 60 with different durations (no quarantine: solid curves; 30 days: dashed curves; 60 days: dashed-dotted curves; 90 days: dotted curves)

Consider first the case of a quarantine that starts at a relatively early stage of the pandemic. Figure 8 illustrates the pandemic dynamics in the absence of a quarantine (solid curves) and on day 60 of the pandemic with different durations.

The duration of a quarantine that starts relatively early, e.g. on day 60, pushes the dynamics forward, about 1.6 days per extra quarantine day, but has hardly any impact on the peak level of infectious individuals and the share of recovered persons after the pandemic has ended (see Table 1). Naturally a longer quarantine is associated with higher economic losses, about 0.1 percentage points for every extra day, as shown in the table below, which presents the $I_{Peak}$-level, the day when this peak is reached, the share of the population that will have been infected and survived, economic output and economic losses for quarantines of different durations. For example, Q60–74 indicates a quarantine starting on day 60 and ending on day 74.

**Table 1:** Outcomes of quarantines of different durations, covering 80 percent of the population and starting on day 60

	${I}_{\mathrm {Peak}}$	${t}({I}_{\mathrm {Peak}})$	R(365)	Y	dY/Y (%)
*No quarantine*	*0.230*	*118*	*0.928*	*359.28*	*– 1.84*
Q60–74	0.223	144	0.925	353.34	– 3.46
Q60–89	0.220	169	0.924	347.38	– 5.09
Q60–104	0.219	194	0.924	341.41	– 6.72
Q60–119	0.218	217	0.924	335.44	– 8.35
Q60–149	0.218	260	0.921	323.49	– 11.61

A quarantine starting on the same day, but covering a smaller share of the population yields different results with respect to the duration. In particular, a smaller share in isolation will impact substantially on the peak level, while having a smaller effect on the peak day and naturally leading to smaller economic losses (see “Extent of the quarantine”).

**Fig. 9:**
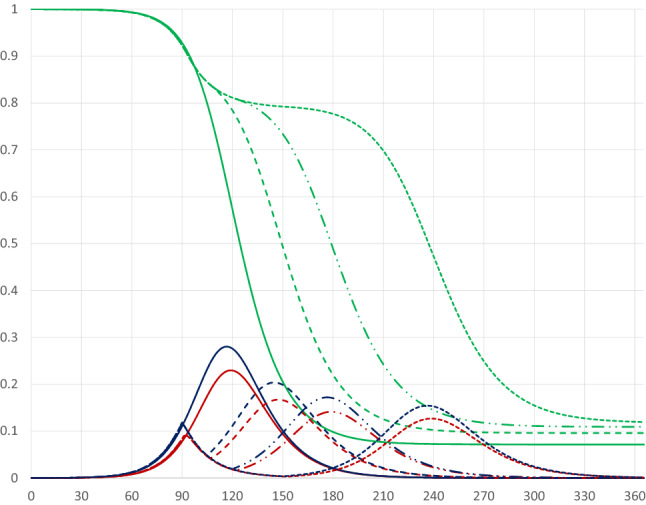
Pandemic dynamics for quarantines covering 80 percent of the population starting on day 90 with different durations (no quarantine: solid curves; 15 days: dashed curves; 30 days: dashed-dotted curves; 60 days: dotted curves)

The impact of the duration of quarantines covering 80 percent of the population is somewhat different when these start at a later stage, e.g. on day 90 of the pandemic, as illustrated in Fig. 9.

**Fig. 10:**
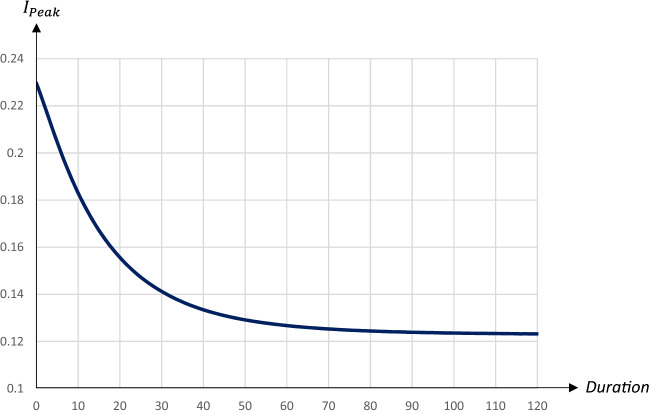
The peak level of infectious people in relation to the duration of a quarantine starting on day 90 and covering 80percent of the population

**Fig. 11:**
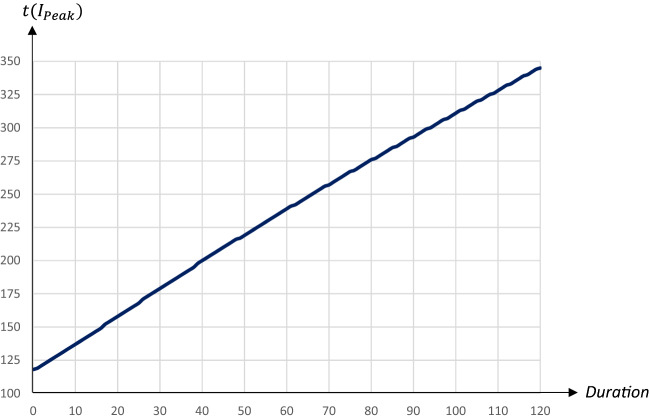
The day of the peak level of infectious people in relation to the duration of a quarantine starting on day 90 and covering 80 percent of the population

All quarantines starting on day 90 lead to a double-peak in the share of infectious individuals, with the first peak occurring on day 90. The second, larger peak is postponed by around two days per extra quarantine day. The $I_{Peak}$-level as well as the share of recovered individuals and deaths decrease in the duration of the quarantine. Figures 10 and 11 illustrate the impact of the duration (the number of days) of a quarantine on the peak level of infectious individuals and the day of the peak occurring.

**Fig. 12:**
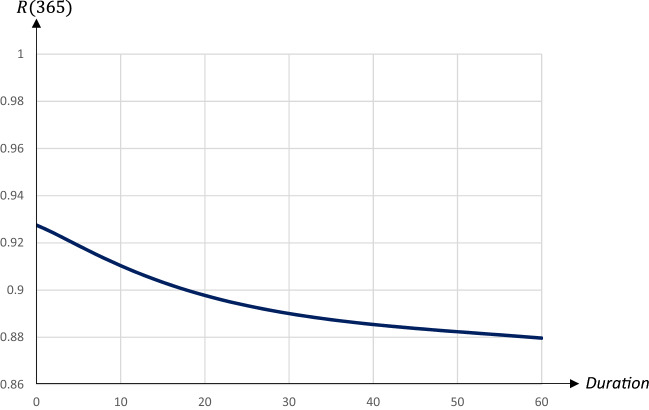
The share of population that has recovered one year after the start of the pandemic in relation to the duration of a quarantine starting on day 90 and covering 80 percent of the population

**Fig. 13:**
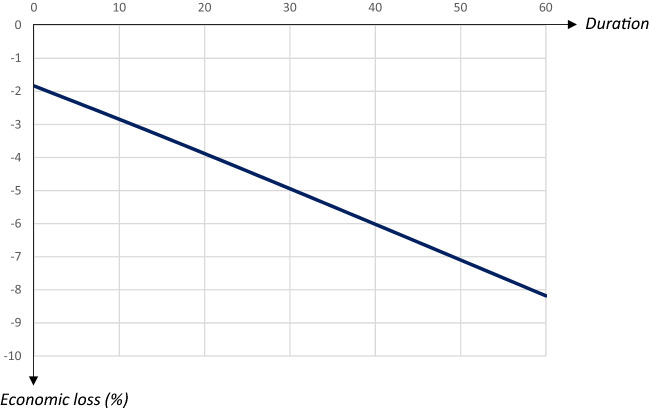
Economic losses in relation to duration of a quarantine starting on day 90 and covering 80 percent of the population

While the peak day is almost linearly related to the duration, the peak level decreases at a decreasing rate in the duration. A quarantine lasting about 30 days reduces the peak level substantially; extending the quarantine beyond 30 days only marginally reduces the peak level, but pushes the peak date forward. Figures 12 and 13 illustrate the impact on the share of recovered individuals after one year and the economic losses in relation to the duration; since quarantines starting on day 90 and lasting more than 60 days lead to the pandemic not having ended after 1 years time, only the effects for durations up to 60 days are presented.

The share of recovered individuals and hence, also the share of deceased persons is only marginally affected by the duration, whereas economic losses increase almost linearly in the duration, by more than 0.1 percentage points for every extra quarantine day.

To summarize, longer quarantines imply larger economic losses. The main effect of a longer duration of a quarantine that is implemented at an early stage of the pandemic is to postpone it. For quarantines that start later the peak level of infectious individuals is reduced by a longer duration, and so is the total number of infected and dead individuals. Thus, there is a relatively clear trade-off between economic costs and health outcomes in terms of the duration of a quarantine.

#### Extent of the quarantine

Finally, we vary the share of the population that is covered by the quarantine, *q*. Again we distinguish between quarantines starting early on and those starting later during the pandemic.

**Fig. 14:**
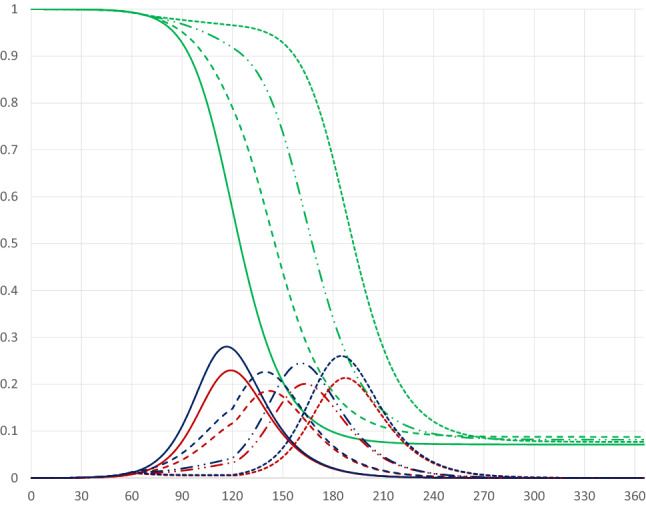
Pandemic dynamics for 60 day quarantines starting on day 60 and covering different shares of the population (no quarantine: solid curves; 20 percent: dashed curves; 40 percent: dashed-dotted curves; 60 percent: dotted curves)

First, we consider quarantines starting relatively early, e.g. on day 60, and lasting for 60 days. Figure 14 illustrates the pandemic dynamics in the absence of a quarantine as well as for quarantines covering different shares of the population.

An increase in the share of quarantined persons unambiguously pushes the $I_{\text {Peak}}$-day forward. The $I_{\text {Peak}}$-level first decreases somewhat, but eventually increases as a higher share of the population is covered by the quarantine. The share of the population having been infected and recovered remains stable above 90 percent. Table 2 summarizes results of simulations; a quarantine covering the entire population is obviously not realistic, but can be used as a benchmark.

**Table 2:** Outcomes of quarantines of different extents, starting on day 60 and lasting for 60 days

	${I}_{\mathrm {Peak}}$	${t}(I_{\mathrm {Peak}})$	R(365)	Y	dY/Y (%)
*No quarantine*	*0.230*	*118*	*0.928*	*359.28*	*– 1.84*
q$=$0.2	0.186	142	0.911	353.43	– 3.44
q$=$0.4	0.201	163	0.917	347.41	– 5.08
q$=$0.6	0.213	187	0.922	341.41	– 6.72
q$=$0.8	0.218	217	0.924	335.44	– 8.35
q$=$1.0	0.219	232	0.924	329.46	– 9.98

Thus, the main effect of increasing *q* for an early quarantine is to push the infection forward in time, but this is associated with substantial economic costs.

**Fig. 15:**
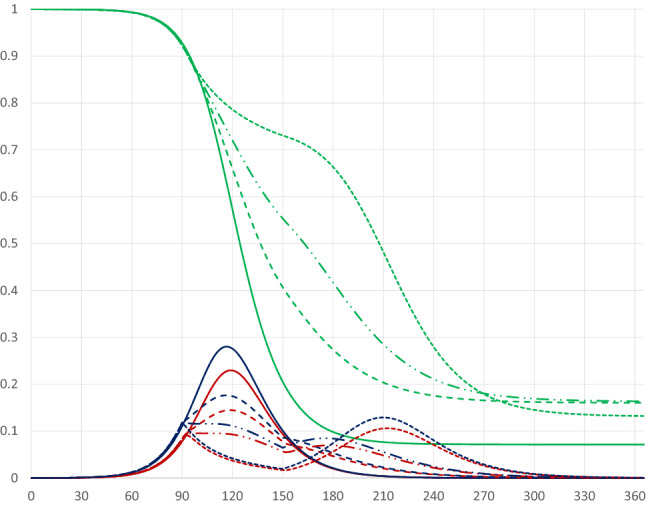
Pandemic dynamics for 60 day quarantines starting on day 90 and covering different shares of the population (no quarantine: solid curves; 20 percent: dashed curves; 35 percent: dashed-dotted curves; 60 percent: dotted curves)

For quarantines starting at a later stage of the pandemic the pattern is slightly different. Figure 15 illustrates the pandemic dynamics in the absence of a quarantine as well as for quarantines starting on day 90 of the pandemic, lasting for 60 days and covering different shares of the population.

**Fig. 16:**
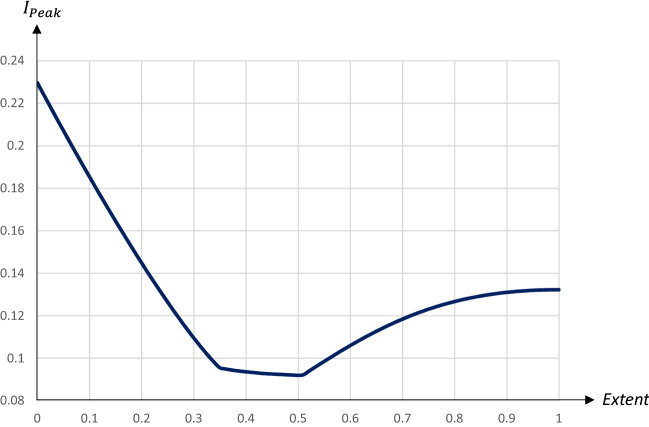
The peak level of infectious people in relation to the extent of a 60 days quarantine starting on day 90

**Fig. 17:**
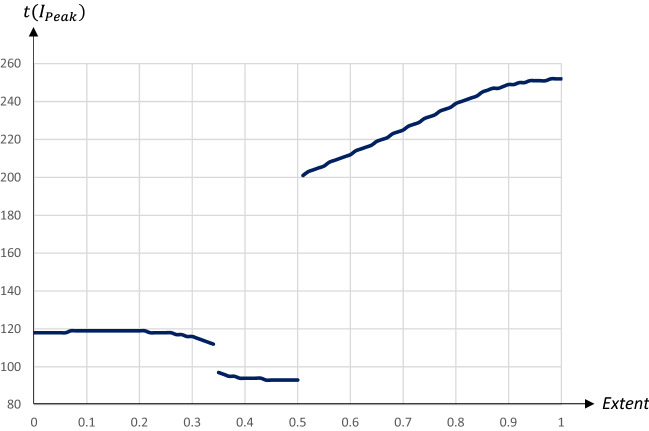
The day of the peak level of infectious people in relation to the extent of a 60 days quarantine starting on day 90

A smaller share of quarantined individuals leads to flatter pandemic dynamics compared to the absence of a quarantine; in particular, the $I_{\text {Peak}}$-level is reduced substantially. For larger shares of quarantined individuals we obtain the familiar double-peak pattern, with the first peak occurring at the starting day of the quarantine. The second peak is actually lower for $q=0.35$ than for $q=0.6$, as a higher share will already have become infectious once the quarantine is terminated. Figures 16 and 17 illustrate how the $I_{\text {Peak}}$-level and the $I_{\text {Peak}}$-day are affected by the extent of the quarantine.

**Fig. 18:**
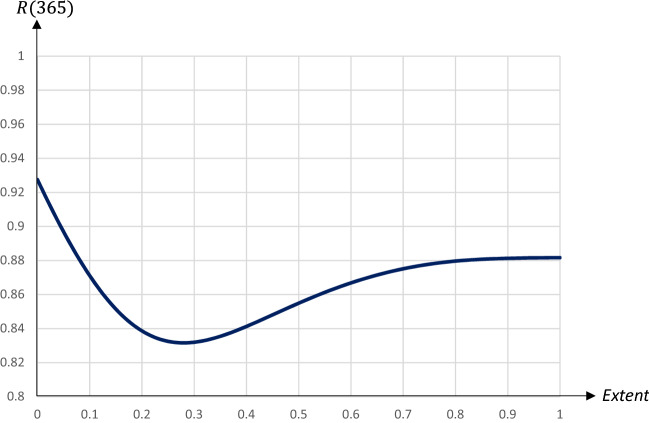
The share of population that has recovered 1 year after the start of the pandemic in relation to the extent of a 60 day quarantine starting on day 90

**Fig. 19:**
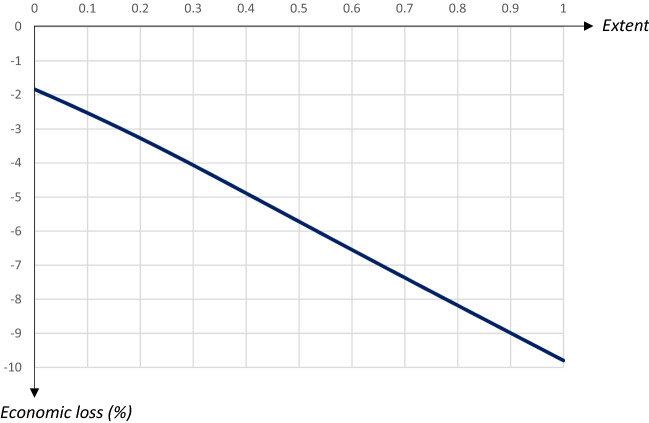
Economic losses in relation to the extent of a 60 day quarantine starting on day 90

An increase in *q* initially reduces the $I_{\text {Peak}}$-level and has only a minor impact on the $I_{\text {Peak}}$-day.^13^ Eventually an increase in *q* brings about the double-peaked pandemic pattern. A higher *q* is associated with an increase in the $I_{\text {Peak}}$-day, but also an increase in the $I_{\text {Peak}}$-level. The impact on the $I_{\text {Peak}}$-level is thus U-shaped, with a minimum reached for $q=0.5$ when the two peaks reach almost the same level.^14^ Figures 18 and  19 illustrate how the share of recovered individuals after one year and economic losses are affected by the extent of the quarantine.

The relationship between the share of recovered (and hence, also deceased) people after the pandemic and the extent of the quarantine is also U-shaped. More specifically, the number of deceased individuals is minimized for $q=0.28$. Economic losses increase almost linearly in the share of quarantined persons, by almost 0.08 percentage points for every extra percent being quarantined.

For $q\in \left[ 0.28,0.5\right]$ there is a trade-off between lowering the $I_{\text {Peak}}$-level on the one hand and reducing the share of people that will have become exposed to the virus on the other hand. If the main goal is to minimize fatalities, a quarantine covering a smaller share of the population is optimal, while a quarantine covering almost half the population is preferable if the aim is to reduce the $I_{\text {Peak}}$-level.

To summarize, the main effect of increasing the share of the quarantined population when the quarantine starts at a relatively early stage of the pandemic is essentially that the peak infection day is pushed forward, but this comes at a substantial economic cost. A quarantine starting at a later stage, when the number of infectious individuals starts increasing rapidly, is associated with a U-shaped relationship between the peak level of infectious individuals and the extent of the quarantine, such that the peak level is reduced substantially for quarantines covering about half the population. At higher *q*-levels the peak is pushed forward, but this also leads to a higher peak level and higher economic losses. The share of deceased people is minimized for quarantines covering a rather small share of the population. Thus, there is a relatively strong case for limiting the extent of a quarantine, since this leads to a lower peak, fewer deaths and lower economic costs. However, such a policy would lead to an earlier peak of infectious people.

## Conclusions

This paper considers some of the basic trade-offs between health outcomes and economic outcomes when a quarantine is implemented. For this purpose we employ a SEIR-model, parameterized to resemble the COVID-19 pandemic and coupled with the assumption that infected and quarantined individuals lose part of their productivity.

Our main findings can be summarized as follows. First, the implementation of an early quarantine postpones but does not alter the course of the pandemic at a cost that increases in the duration and the extent of the quarantine. Second, a quarantine implemented when the number of infectious persons starts increasing rapidly is optimal if the focus is on reducing the peak level, while a starting day just before the peak of infectious people is reached is optimal if the main goal is to reduce fatalities and economic losses. Third, there is a trade-off between economic costs and health outcomes when it comes to the duration of a quarantine. A longer quarantine either postpones the peak (if it is implemented relatively early) or dampens the peak and reduces deaths (if it starts at a later stage of the pandemic), but implies higher economic losses. Finally, there is a relatively strong case for limiting the extent of a quarantine. A less than complete quarantine leads to a lower peak, fewer deaths and lower economic costs. The flip side of this strategy is that the peak of infectious individuals occurs earlier.

To test the robustness of our results we have simulated pandemics with both higher and lower transmission rates. Qualitatively all our findings can be replicated for different pandemic dynamics. Thus, the conclusions regarding the timing, duration and extent of quarantines hold generally in the.context of our model.

## Footnotes

1 This type of epidemiological model was introduced by Kermack and McKendrick [14].

2 See He et al. [12].

3 This relatively optimistic value for $\delta$ is consistent with the study by Bendavid et al. [6]. However, the choice of $\delta$ has virtually no effect on the infection dynamics.

4 This value of $\beta$ is used by e.g. Alvarez et al. [1].

5 The share of daily new cases of infectious people peaks at 1.97 percent on day 109.

6 The infection here has, for practical purposes, run its course after 365 days even without policy interventions. However, because population is continuous in this type of model there is always a very small share still infected. This is also true after quarantines are imposed. In reality the infection would be completely eradicated once the last infected person is either recovered or dead.

7 After the second day of the quarantine the share of daily new cases of infectious people starts dropping from 1.16 percent to 0.01 percent within a week.

8 This case is discussed by Anderson et al. [2].

9 In this case the number of newly infectious people per day has already started falling when the quarantine is implemented. This decline is accelerated, such that the share of daily new cases of infectious persons decreases from 1.55 percent to 0.02 percent within a week.

10 For a 30 days quarantine covering only 20 percent of the population the $I_{Peak}$-level would reach its minimum if it is started on day 93; however, the minimum $I_{Peak}$-level would be somewhat higher, at 0.15. For a 60 days quarantine covering 80 percent of individuals the $I_{Peak}$ -level would also reach its minimum if implemented on day 93; in this case the minimum $I_{Peak}$-level would be somewhat lower, at 0.1086.

11 The herd immunity threshold would be about 64% of the population given that $R_{0}=2.8$.

12 For a 30 days quarantine covering only 20 percent of the population the *R*(365)-value would be larger, reaching its minimum for one started on day 109 (at 0.875), while economic losses would be smaller, being minimized for a quarantine starting on day 111. For a 60 days quarantine covering 80percent of individuals the *R*(365) -value would be smaller and minimized for one implemented on day 111 (at 0.668), while economic losses would be larger, being minimized for a quarantine starting on day 112.

13 Note that for quarantines covering around a third of the population the first peak resembles a plateau lasting for almost 30 days (see Fig. 15 when $q=0.35$). We, therefore, observe a drop in the peak-level day when *q* increases from 0.34 to 0.35, as the peak of this plateau shifts from day 112 to day 97.

14 As the extent of the quarantine increases above $q=0.5$, the second peak becomes higher than the first peak, such that the day when the peak-level of infectious persons occurs jumps from 93 to 201.
